# [3-Meth­oxy-1-(phenyl­sulfan­yl)prop­yl]triphenyl­tin(IV) benzene 0.17-solvate

**DOI:** 10.1107/S1600536811055474

**Published:** 2012-01-14

**Authors:** Gerd Ludwig, Michael Block, Christoph Wagner, Dirk Steinborn

**Affiliations:** a Institut für Chemie – Anorganische Chemie, Martin-Luther-Universität Halle-Wittenberg, D-06120 Halle, Kurt-Mothes-Strasse 2, Germany

## Abstract

In the title compound, [Sn(C_6_H_5_)_3_(C_10_H_13_OS)]·0.17C_6_H_6_, the Sn^IV^ atom exhibits a slightly distorted tetra­hedral coordination geometry built up by four C atoms, which are the three *ipso*-C atoms of the phenyl rings and the α-C atom of the deprotonated γ-*O*-functionalized propyl phenyl sulfide. The benzene mol­ecule lies about a threefold rotoinversion axis.

## Related literature

The synthesis of the tin compound was performed according to Block *et al.* (2009 ▶). For a better understanding of the use and synthesis of heteroatom-functionalized tin compounds, see: Kauffmann *et al.* (1982 ▶); Linnert *et al.* (2008 ▶).
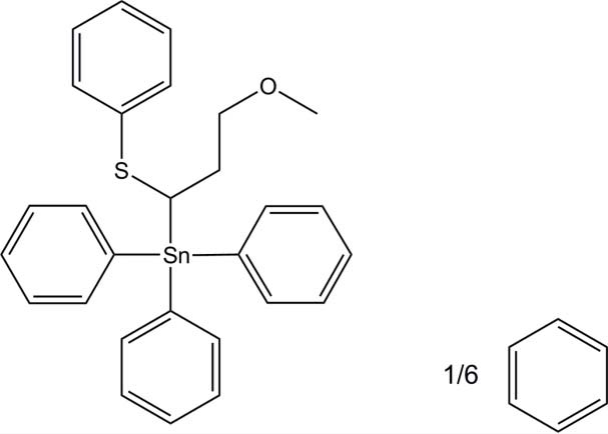


## Experimental

### 

#### Crystal data

[Sn(C_6_H_5_)_3_(C_10_H_13_OS)]·0.17C_6_H_6_
*M* *_r_* = 544.27
Trigonal, 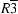

*a* = 35.338 (3) Å
*c* = 10.5660 (8) Å
*V* = 11427.1 (16) Å^3^
*Z* = 18
Mo *K*α radiation
μ = 1.11 mm^−1^
*T* = 220 K
0.46 × 0.31 × 0.15 mm

#### Data collection

Stoe IPDS diffractometer
Absorption correction: numerical (*IPDS Software*; Stoe & Cie, 1999 ▶) *T*_min_ = 0.648, *T*_max_ = 0.847
22832 measured reflections
4905 independent reflections
4008 reflections with *I* > 2σ(*I*)
*R*_int_ = 0.061

#### Refinement

*R*[*F* ^2^ > 2σ(*F* ^2^)] = 0.029
*wR*(*F* ^2^) = 0.065
*S* = 0.99
4905 reflections
288 parameters
1 restraint
H-atom parameters constrained
Δρ_max_ = 0.57 e Å^−3^
Δρ_min_ = −0.53 e Å^−3^

### 

Data collection: *IPDS* *EXPOSE* (Stoe & Cie, 1999 ▶); cell refinement: *IPDS* *EXPOSE*; data reduction: *IPDS* *INTEGRATE* (Stoe & Cie, 1999 ▶); program(s) used to solve structure: *SHELXS97* (Sheldrick, 2008 ▶); program(s) used to refine structure: *SHELXL97* (Sheldrick, 2008 ▶); molecular graphics: *DIAMOND* (Brandenburg, 2001 ▶); software used to prepare material for publication: *SHELXL97*.

## Supplementary Material

Crystal structure: contains datablock(s) global, I. DOI: 10.1107/S1600536811055474/is5011sup1.cif

Structure factors: contains datablock(s) I. DOI: 10.1107/S1600536811055474/is5011Isup2.hkl

Additional supplementary materials:  crystallographic information; 3D view; checkCIF report

## Figures and Tables

**Table 1:** Selected bond lengths (Å)

C1—Sn	2.196 (2)
C5—S	1.774 (3)
C11—Sn	2.150 (2)
C17—Sn	2.153 (2)
C23—Sn	2.147 (2)

**Fig. 1.**
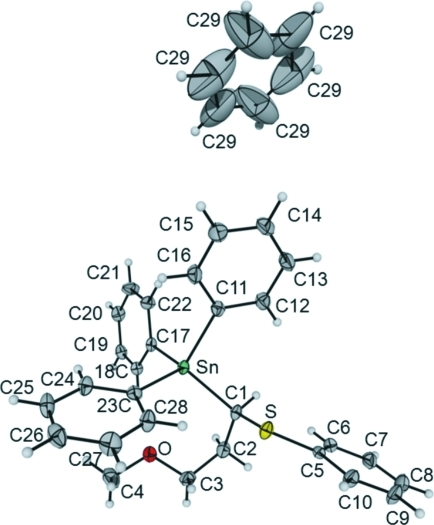
Structure of the title compound. Displacement ellipsoids are drawn at the 30% probability level and the H atoms are shown as small spheres of arbitrary radii.

## supplementary crystallographic information

### Comment

The synthesis of the tin compound was performed according to Block *et al.* (2009). The title compound, [Sn(C_6_H_5_)_3_(C_10_H_13_OS)].0.17C_6_H_6_, crystallizes in the trigonal crystal system in the space group *R*–3. In crystals isolated molecules of the tin compound and of the solvent molecules (C_6_H_6_) were found. No unusual intermolecular interactions exist between them; the shortest distance between non-hydrogen atoms is 3.540 (5) Å [C12···C16(1/3 + *x* - *y*, -1/3 + *x*, 5/3 - *z*)]. The molecular structure of the tin compound is shown in Figure 1, selected bond lengths and angles are summarized in Table 1. The primary coordination sphere of the tin atom is built up by four carbon atoms, which are the three *ipso*-C atoms of the phenyl rings and the α-C atom of the deprotonated γ-*O*-functionalized propyl phenyl sulfide. Thus, the presence of an α-stannylated sulfide could be clearly proved. The C1–S–C5 angle is 105.7 (1)°; therefore the C1 atom has to be described as *sp*^3^ hybridized. The configuration of the tin atom is slightly tetrahedral distorted; the C–Sn–C angles range from 103.8 (9) to 114.57 (9)°. The distance between the tin atom and the oxygen atom of the pending methoxy group is 3.100 (2) Å which points to a weak intramolecular interaction between these two atoms. The center of the solvent molecules (C_6_H_6_) has -3 site symmetry. Thus, the asymmetric unit contains only one CH group.

### Experimental

At -78 °C, to a solution of *n*-BuLi in *n*-hexane (1.5 *M*, 0.01 mol) in toluene (20 ml), TMEDA (0.01 mol) was added and, after stirring for 15 min, PhSCH_2_CH_2_CH_2_OCH_3_ (0.01 mol). Then, the reaction mixture was stirred for 24 h at room temperature. This was followed by dropwise addtion of Ph_3_SnCl (0.01 mol) at -78 °C and by stirring for 24 h at room temperature. Then, the reaction mixture was treated with a saturated solution of NH_4_Cl in water (50 ml). The aqueous phase was washed with diethyl ether (3 × 30 ml). The combined organic phases were dried (Na_2_SO_4_), the solvents were removed *in vacuo* and the crude product was purified by centrifugally accelerated thin layer chromatography with diethyl ether as eluent (yield 3.98 g, 76%).Single crystals suitable for X-ray diffraction measurements were obtained by recrystallization from benzene. Characterization: ^1^H-NMR (500 MHz, CDCl_3_): δ 2.19 – 2.25 (m, 2H, CH_2_C*H*_2_CH), 2.89 (s, 3H, OC*H*_3_), 3.01 – 3.04 (m, 1H, SnC*H*), 3.36 – 3.69 (m, 2H, C*H*_2_OCH_3_), 7.18 – 7.19 (m, 1H, *p*-*H*, SPh), 7.25 – 7.36 (m, 2H, *m*-*H*, SPh), 7.37 – 7.41 (m, 21H, C_6_*H*_5_, Sn(Ph)_3_ + C_6_H_6_), 7.60 – 7.66 (m, 2H, *o*-*H*, SPh).^119^Sn-NMR (186 MHz, CDCl_3_): δ 126.3 (*s*).

### Refinement

All H atoms were positioned geometrically and treated as riding model, with C—H bond distances of 0.98, 0.99 and 1.00 Å for CH_3_, CH_2_ and CH type H-atoms, respectively, and with *U*_iso_(H) = 1.5 times *U*_eq_(methyl C) and 1.2 times *U*_eq_(non-methyl C). To modify the C—C bond lengths of the benzene molecule the restraint C—C = 1.390 (5) Å was used.

### Crystal data

**Table:**

[Sn(C_6_H_5_)_3_(C_10_H_13_OS)]·0.17C_6_H_6_	*D*_x_ = 1.424 Mg m^−^^3^
*M**_r_* = 544.27	Mo *K*α radiation, λ = 0.71073 Å
Trigonal, *R*3	Cell parameters from 8000 reflections
Hall symbol: -R 3	θ = 2.0–25.6°
*a* = 35.338 (3) Å	µ = 1.11 mm^−^^1^
*c* = 10.5660 (8) Å	*T* = 220 K
*V* = 11427.1 (16) Å^3^	Block, colourless
*Z* = 18	0.46 × 0.31 × 0.15 mm
*F*(000) = 4986	

### Data collection

**Table:**

Stoe IPDS diffractometer	4905 independent reflections
Radiation source: fine-focus sealed tube	4008 reflections with *I* > 2σ(*I*)
graphite	*R*_int_ = 0.061
area detector scans	θ_max_ = 25.9°, θ_min_ = 2.0°
Absorption correction: numerical (*IPDS Software*; Stoe & Cie, 1999)	*h* = −43→43
*T*_min_ = 0.648, *T*_max_ = 0.847	*k* = −43→42
22832 measured reflections	*l* = −12→12

### Refinement

**Table:**

Refinement on *F*^2^	Primary atom site location: structure-invariant direct methods
Least-squares matrix: full	Secondary atom site location: difference Fourier map
*R*[*F*^2^ > 2σ(*F*^2^)] = 0.029	Hydrogen site location: inferred from neighbouring sites
*wR*(*F*^2^) = 0.065	H-atom parameters constrained
*S* = 0.99	*w* = 1/[σ^2^(*F*_o_^2^) + (0.0371*P*)^2^] where *P* = (*F*_o_^2^ + 2*F*_c_^2^)/3
4905 reflections	(Δ/σ)_max_ = 0.002
288 parameters	Δρ_max_ = 0.57 e Å^−^^3^
1 restraint	Δρ_min_ = −0.52 e Å^−^^3^

### Special details

**Table:**

Geometry. All e.s.d.'s (except the e.s.d. in the dihedral angle between two l.s. planes) are estimated using the full covariance matrix. The cell e.s.d.'s are taken into account individually in the estimation of e.s.d.'s in distances, angles and torsion angles; correlations between e.s.d.'s in cell parameters are only used when they are defined by crystal symmetry. An approximate (isotropic) treatment of cell e.s.d.'s is used for estimating e.s.d.'s involving l.s. planes.
Refinement. Refinement of *F*^2^ against ALL reflections. The weighted *R*-factor *wR* and goodness of fit *S* are based on *F*^2^, conventional *R*-factors *R* are based on *F*, with *F* set to zero for negative *F*^2^. The threshold expression of *F*^2^ > σ(*F*^2^) is used only for calculating *R*-factors(gt) *etc*. and is not relevant to the choice of reflections for refinement. *R*-factors based on *F*^2^ are statistically about twice as large as those based on *F*, and *R*- factors based on ALL data will be even larger.

### Fractional atomic coordinates and isotropic or equivalent isotropic displacement parameters (Å^2^)

**Table:**

	*x*	*y*	*z*	*U*_iso_*/*U*_eq_	
C1	0.46298 (8)	0.12671 (8)	0.9677 (2)	0.0295 (5)	
H1	0.4833	0.1173	0.9386	0.035*	
C2	0.42345 (9)	0.08653 (9)	1.0248 (3)	0.0421 (7)	
H3	0.4134	0.0627	0.9645	0.050*	
H2	0.4330	0.0779	1.1000	0.050*	
C3	0.38543 (9)	0.09245 (10)	1.0599 (3)	0.0460 (7)	
H5	0.3647	0.0680	1.1110	0.055*	
H4	0.3955	0.1191	1.1085	0.055*	
C4	0.33026 (10)	0.10298 (12)	0.9689 (4)	0.0637 (9)	
H6	0.3176	0.1040	0.8895	0.076*	
H8	0.3412	0.1305	1.0117	0.076*	
H7	0.3085	0.0801	1.0204	0.076*	
C5	0.51631 (8)	0.14921 (9)	1.1889 (2)	0.0326 (6)	
C6	0.51916 (8)	0.11198 (9)	1.1672 (2)	0.0364 (6)	
H9	0.5065	0.0952	1.0951	0.044*	
C7	0.54086 (9)	0.09969 (10)	1.2528 (3)	0.0479 (7)	
H10	0.5426	0.0747	1.2378	0.057*	
C8	0.55965 (10)	0.12407 (13)	1.3589 (3)	0.0599 (9)	
H11	0.5745	0.1159	1.4153	0.072*	
C9	0.55662 (11)	0.16073 (13)	1.3820 (3)	0.0639 (10)	
H12	0.5693	0.1771	1.4546	0.077*	
C10	0.53482 (9)	0.17353 (10)	1.2982 (3)	0.0468 (7)	
H13	0.5326	0.1982	1.3149	0.056*	
C11	0.51525 (8)	0.20375 (8)	0.7377 (2)	0.0314 (5)	
C12	0.55158 (9)	0.19985 (9)	0.7660 (3)	0.0414 (6)	
H14	0.5480	0.1765	0.8153	0.050*	
C13	0.59285 (10)	0.22978 (10)	0.7227 (3)	0.0519 (8)	
H15	0.6167	0.2264	0.7424	0.062*	
C14	0.59838 (10)	0.26436 (11)	0.6508 (3)	0.0576 (9)	
H16	0.6261	0.2847	0.6217	0.069*	
C15	0.56311 (11)	0.26918 (11)	0.6216 (4)	0.0666 (10)	
H17	0.5670	0.2928	0.5730	0.080*	
C16	0.52209 (9)	0.23920 (9)	0.6638 (3)	0.0486 (8)	
H18	0.4985	0.2427	0.6426	0.058*	
C17	0.41851 (8)	0.11558 (8)	0.6483 (2)	0.0272 (5)	
C18	0.38006 (8)	0.07525 (8)	0.6555 (2)	0.0323 (6)	
H19	0.3660	0.0654	0.7330	0.039*	
C19	0.36252 (9)	0.04966 (9)	0.5481 (3)	0.0370 (6)	
H20	0.3370	0.0227	0.5544	0.044*	
C20	0.38254 (9)	0.06382 (10)	0.4326 (3)	0.0425 (7)	
H21	0.3706	0.0465	0.3611	0.051*	
C21	0.42028 (10)	0.10362 (10)	0.4230 (3)	0.0496 (7)	
H22	0.4337	0.1135	0.3448	0.060*	
C22	0.43828 (9)	0.12899 (9)	0.5303 (2)	0.0389 (6)	
H23	0.4642	0.1556	0.5232	0.047*	
C23	0.41951 (8)	0.19495 (8)	0.8547 (2)	0.0303 (5)	
C24	0.38656 (9)	0.19384 (9)	0.7791 (3)	0.0392 (6)	
H24	0.3785	0.1775	0.7049	0.047*	
C25	0.36561 (9)	0.21676 (10)	0.8130 (3)	0.0488 (8)	
H25	0.3439	0.2158	0.7612	0.059*	
C26	0.37677 (10)	0.24078 (10)	0.9222 (3)	0.0515 (8)	
H26	0.3624	0.2557	0.9452	0.062*	
C27	0.40953 (11)	0.24266 (10)	0.9980 (3)	0.0515 (8)	
H27	0.4174	0.2590	1.0721	0.062*	
C28	0.43065 (9)	0.22026 (9)	0.9638 (3)	0.0411 (6)	
H28	0.4529	0.2222	1.0151	0.049*	
C29	0.6516 (4)	0.28958 (16)	0.3333	0.240 (8)	
H29	0.6412	0.2597	0.3333	0.288*	
O	0.36531 (6)	0.09476 (7)	0.94676 (18)	0.0436 (5)	
S	0.49207 (2)	0.16969 (2)	1.08222 (6)	0.03528 (15)	
Sn	0.450886 (5)	0.158288 (5)	0.805962 (15)	0.02691 (7)	

### Atomic displacement parameters (Å^2^)

**Table:**

	*U*^11^	*U*^22^	*U*^33^	*U*^12^	*U*^13^	*U*^23^
C1	0.0326 (13)	0.0278 (13)	0.0288 (12)	0.0156 (11)	−0.0092 (10)	−0.0061 (10)
C2	0.0369 (16)	0.0352 (15)	0.0466 (16)	0.0124 (13)	−0.0094 (12)	0.0065 (12)
C3	0.0356 (16)	0.0497 (18)	0.0361 (15)	0.0090 (14)	0.0010 (12)	0.0098 (13)
C4	0.0358 (18)	0.062 (2)	0.090 (3)	0.0214 (16)	0.0102 (16)	0.0190 (19)
C5	0.0211 (12)	0.0434 (15)	0.0255 (12)	0.0102 (11)	0.0017 (9)	0.0030 (10)
C6	0.0293 (14)	0.0427 (16)	0.0348 (14)	0.0163 (12)	−0.0009 (11)	0.0041 (11)
C7	0.0363 (16)	0.0532 (19)	0.0528 (18)	0.0214 (15)	0.0000 (13)	0.0169 (14)
C8	0.0445 (19)	0.075 (2)	0.0509 (19)	0.0233 (18)	−0.0127 (15)	0.0145 (17)
C9	0.058 (2)	0.082 (3)	0.0319 (16)	0.0205 (19)	−0.0189 (14)	−0.0066 (15)
C10	0.0459 (17)	0.0558 (19)	0.0315 (15)	0.0199 (15)	−0.0080 (12)	−0.0050 (13)
C11	0.0300 (13)	0.0280 (13)	0.0336 (13)	0.0125 (11)	−0.0039 (10)	−0.0024 (10)
C12	0.0377 (16)	0.0354 (15)	0.0548 (17)	0.0210 (13)	0.0004 (13)	0.0060 (13)
C13	0.0337 (16)	0.054 (2)	0.071 (2)	0.0250 (15)	0.0023 (14)	0.0104 (16)
C14	0.0305 (16)	0.0508 (19)	0.077 (2)	0.0095 (14)	0.0039 (15)	0.0191 (17)
C15	0.047 (2)	0.052 (2)	0.093 (3)	0.0186 (17)	0.0033 (18)	0.0370 (19)
C16	0.0344 (16)	0.0430 (17)	0.068 (2)	0.0191 (14)	−0.0056 (14)	0.0145 (14)
C17	0.0274 (13)	0.0287 (13)	0.0296 (12)	0.0171 (11)	−0.0063 (10)	−0.0038 (10)
C18	0.0302 (14)	0.0347 (14)	0.0336 (13)	0.0176 (12)	−0.0006 (10)	0.0007 (11)
C19	0.0298 (14)	0.0330 (14)	0.0455 (16)	0.0137 (12)	−0.0071 (11)	−0.0076 (12)
C20	0.0434 (16)	0.0478 (17)	0.0387 (15)	0.0245 (14)	−0.0101 (12)	−0.0176 (13)
C21	0.0495 (18)	0.058 (2)	0.0286 (15)	0.0178 (16)	0.0029 (12)	−0.0052 (13)
C22	0.0351 (15)	0.0398 (16)	0.0319 (14)	0.0114 (12)	−0.0002 (11)	−0.0019 (11)
C23	0.0284 (13)	0.0250 (13)	0.0347 (13)	0.0114 (11)	0.0012 (10)	0.0034 (10)
C24	0.0380 (15)	0.0362 (15)	0.0436 (15)	0.0186 (13)	−0.0100 (12)	−0.0035 (12)
C25	0.0374 (16)	0.0437 (17)	0.072 (2)	0.0250 (14)	−0.0152 (14)	−0.0006 (15)
C26	0.0517 (19)	0.0409 (17)	0.074 (2)	0.0325 (16)	0.0040 (16)	0.0000 (15)
C27	0.063 (2)	0.0462 (18)	0.0528 (18)	0.0328 (16)	−0.0086 (15)	−0.0151 (14)
C28	0.0457 (17)	0.0374 (15)	0.0468 (16)	0.0257 (14)	−0.0118 (12)	−0.0081 (12)
C29	0.131 (6)	0.323 (12)	0.148 (6)	0.026 (10)	0.042 (5)	−0.081 (9)
O	0.0355 (11)	0.0527 (12)	0.0421 (11)	0.0217 (10)	−0.0037 (8)	0.0044 (9)
S	0.0414 (4)	0.0333 (3)	0.0324 (3)	0.0197 (3)	−0.0108 (3)	−0.0076 (3)
Sn	0.02727 (10)	0.02668 (10)	0.02675 (10)	0.01346 (8)	−0.00504 (6)	−0.00283 (6)

### Geometric parameters (Å, °)

**Table:**

C1—C2	1.532 (4)	C14—C15	1.375 (5)
C1—S	1.807 (2)	C14—H16	0.9300
C1—Sn	2.196 (2)	C15—C16	1.374 (4)
C1—H1	0.9800	C15—H17	0.9300
C2—C3	1.506 (4)	C16—H18	0.9300
C2—H3	0.9700	C17—C22	1.392 (3)
C2—H2	0.9700	C17—C18	1.395 (3)
C3—O	1.414 (3)	C17—Sn	2.153 (2)
C3—H5	0.9700	C18—C19	1.389 (4)
C3—H4	0.9700	C18—H19	0.9300
C4—O	1.426 (4)	C19—C20	1.373 (4)
C4—H6	0.9600	C19—H20	0.9300
C4—H8	0.9600	C20—C21	1.375 (4)
C4—H7	0.9600	C20—H21	0.9300
C5—C6	1.388 (4)	C21—C22	1.387 (4)
C5—C10	1.391 (4)	C21—H22	0.9300
C5—S	1.774 (3)	C22—H23	0.9300
C6—C7	1.389 (4)	C23—C28	1.390 (4)
C6—H9	0.9300	C23—C24	1.396 (4)
C7—C8	1.367 (5)	C23—Sn	2.147 (2)
C7—H10	0.9300	C24—C25	1.390 (4)
C8—C9	1.374 (5)	C24—H24	0.9300
C8—H11	0.9300	C25—C26	1.368 (4)
C9—C10	1.390 (5)	C25—H25	0.9300
C9—H12	0.9300	C26—C27	1.381 (4)
C10—H13	0.9300	C26—H26	0.9300
C11—C12	1.391 (4)	C27—C28	1.380 (4)
C11—C16	1.391 (4)	C27—H27	0.9300
C11—Sn	2.150 (2)	C28—H28	0.9300
C12—C13	1.382 (4)	C29—C29^i^	1.360 (5)
C12—H14	0.9300	C29—C29^ii^	1.360 (5)
C13—C14	1.368 (4)	C29—H29	0.9300
C13—H15	0.9300		
			
C2—C1—S	112.78 (18)	C16—C15—C14	120.3 (3)
C2—C1—Sn	117.27 (16)	C16—C15—H17	119.9
S—C1—Sn	105.52 (11)	C14—C15—H17	119.9
C2—C1—H1	106.9	C15—C16—C11	121.2 (3)
S—C1—H1	106.9	C15—C16—H18	119.4
Sn—C1—H1	106.9	C11—C16—H18	119.4
C3—C2—C1	115.6 (2)	C22—C17—C18	117.6 (2)
C3—C2—H3	108.4	C22—C17—Sn	117.01 (17)
C1—C2—H3	108.4	C18—C17—Sn	125.37 (18)
C3—C2—H2	108.4	C19—C18—C17	120.7 (2)
C1—C2—H2	108.4	C19—C18—H19	119.7
H3—C2—H2	107.5	C17—C18—H19	119.7
O—C3—C2	108.0 (2)	C20—C19—C18	120.5 (2)
O—C3—H5	110.1	C20—C19—H20	119.7
C2—C3—H5	110.1	C18—C19—H20	119.7
O—C3—H4	110.1	C19—C20—C21	119.8 (2)
C2—C3—H4	110.1	C19—C20—H21	120.1
H5—C3—H4	108.4	C21—C20—H21	120.1
O—C4—H6	109.5	C20—C21—C22	119.9 (3)
O—C4—H8	109.5	C20—C21—H22	120.1
H6—C4—H8	109.5	C22—C21—H22	120.1
O—C4—H7	109.5	C21—C22—C17	121.5 (3)
H6—C4—H7	109.5	C21—C22—H23	119.3
H8—C4—H7	109.5	C17—C22—H23	119.3
C6—C5—C10	119.2 (2)	C28—C23—C24	117.1 (2)
C6—C5—S	124.04 (19)	C28—C23—Sn	121.46 (18)
C10—C5—S	116.7 (2)	C24—C23—Sn	121.40 (19)
C5—C6—C7	120.1 (3)	C25—C24—C23	121.1 (3)
C5—C6—H9	119.9	C25—C24—H24	119.5
C7—C6—H9	119.9	C23—C24—H24	119.5
C8—C7—C6	120.5 (3)	C26—C25—C24	120.4 (3)
C8—C7—H10	119.7	C26—C25—H25	119.8
C6—C7—H10	119.7	C24—C25—H25	119.8
C7—C8—C9	119.8 (3)	C25—C26—C27	119.5 (3)
C7—C8—H11	120.1	C25—C26—H26	120.2
C9—C8—H11	120.1	C27—C26—H26	120.2
C8—C9—C10	120.7 (3)	C28—C27—C26	120.1 (3)
C8—C9—H12	119.6	C28—C27—H27	120.0
C10—C9—H12	119.6	C26—C27—H27	120.0
C9—C10—C5	119.6 (3)	C27—C28—C23	121.7 (3)
C9—C10—H13	120.2	C27—C28—H28	119.1
C5—C10—H13	120.2	C23—C28—H28	119.1
C12—C11—C16	117.2 (2)	C29^i^—C29—C29^ii^	120.006 (1)
C12—C11—Sn	122.56 (19)	C29^i^—C29—H29	120.0
C16—C11—Sn	120.26 (19)	C29^ii^—C29—H29	120.0
C13—C12—C11	121.8 (3)	C3—O—C4	112.7 (2)
C13—C12—H14	119.1	C5—S—C1	105.70 (12)
C11—C12—H14	119.1	C23—Sn—C11	107.59 (9)
C14—C13—C12	119.5 (3)	C23—Sn—C17	110.79 (9)
C14—C13—H15	120.3	C11—Sn—C17	104.90 (9)
C12—C13—H15	120.3	C23—Sn—C1	114.23 (9)
C13—C14—C15	120.2 (3)	C11—Sn—C1	103.82 (9)
C13—C14—H16	119.9	C17—Sn—C1	114.57 (9)
C15—C14—H16	119.9		
			
S—C1—C2—C3	70.6 (3)	C26—C27—C28—C23	−0.9 (5)
Sn—C1—C2—C3	−52.3 (3)	C24—C23—C28—C27	1.4 (4)
C1—C2—C3—O	71.8 (3)	Sn—C23—C28—C27	−177.6 (2)
C10—C5—C6—C7	−1.0 (4)	C2—C3—O—C4	−176.5 (2)
S—C5—C6—C7	176.4 (2)	C6—C5—S—C1	12.6 (2)
C5—C6—C7—C8	−0.1 (4)	C10—C5—S—C1	−170.0 (2)
C6—C7—C8—C9	0.8 (5)	C2—C1—S—C5	68.1 (2)
C7—C8—C9—C10	−0.4 (5)	Sn—C1—S—C5	−162.64 (11)
C8—C9—C10—C5	−0.7 (5)	C28—C23—Sn—C11	−74.8 (2)
C6—C5—C10—C9	1.4 (4)	C24—C23—Sn—C11	106.2 (2)
S—C5—C10—C9	−176.2 (2)	C28—C23—Sn—C17	171.1 (2)
C16—C11—C12—C13	−0.1 (4)	C24—C23—Sn—C17	−7.9 (2)
Sn—C11—C12—C13	−179.0 (2)	C28—C23—Sn—C1	39.9 (2)
C11—C12—C13—C14	0.4 (5)	C24—C23—Sn—C1	−139.1 (2)
C12—C13—C14—C15	−0.2 (6)	C16—C11—Sn—C23	−37.7 (2)
C13—C14—C15—C16	−0.3 (6)	C12—C11—Sn—C23	141.3 (2)
C14—C15—C16—C11	0.7 (6)	C16—C11—Sn—C17	80.3 (2)
C12—C11—C16—C15	−0.5 (5)	C12—C11—Sn—C17	−100.7 (2)
Sn—C11—C16—C15	178.5 (3)	C16—C11—Sn—C1	−159.1 (2)
C22—C17—C18—C19	0.2 (4)	C12—C11—Sn—C1	19.8 (2)
Sn—C17—C18—C19	−178.21 (18)	C22—C17—Sn—C23	101.8 (2)
C17—C18—C19—C20	−0.6 (4)	C18—C17—Sn—C23	−79.8 (2)
C18—C19—C20—C21	0.1 (4)	C22—C17—Sn—C11	−14.0 (2)
C19—C20—C21—C22	0.9 (5)	C18—C17—Sn—C11	164.4 (2)
C20—C21—C22—C17	−1.4 (5)	C22—C17—Sn—C1	−127.14 (19)
C18—C17—C22—C21	0.8 (4)	C18—C17—Sn—C1	51.2 (2)
Sn—C17—C22—C21	179.4 (2)	C2—C1—Sn—C23	73.5 (2)
C28—C23—C24—C25	−0.7 (4)	S—C1—Sn—C23	−52.99 (14)
Sn—C23—C24—C25	178.3 (2)	C2—C1—Sn—C11	−169.59 (19)
C23—C24—C25—C26	−0.4 (5)	S—C1—Sn—C11	63.88 (13)
C24—C25—C26—C27	0.9 (5)	C2—C1—Sn—C17	−55.8 (2)
C25—C26—C27—C28	−0.3 (5)	S—C1—Sn—C17	177.69 (10)

Symmetry codes: (i) *y*+1/3, −*x*+*y*+2/3, −*z*+2/3; (ii) *x*−*y*+1/3, *x*−1/3, −*z*+2/3.
